# Comparison of Surgical Outcomes Between the hinotori™ and da Vinci® Robotic Systems in Robot-Assisted Sacrocolpopexy: A Retrospective Study

**DOI:** 10.7759/cureus.89857

**Published:** 2025-08-12

**Authors:** Shigehisa Kubota, Richi Okusue, Masayuki Nagasawa, Akinori Wada, Kenichi Kobayashi, Kazuaki Yamanaka, Teruhiko Tsuru, Tetsuya Yoshida, Kazuyoshi Johnin, Susumu Kageyama

**Affiliations:** 1 Department of Urology, Shiga University of Medical Science, Otsu, JPN; 2 Department of Urology, Otsu City Hospital, Otsu, JPN

**Keywords:** davinci surgical system, hinotori surgical system, operative time comparison, pelvic organ prolapse (pop), robot-assisted sacrocolpopexy

## Abstract

Objective

This study aimed to assess whether the hinotori™ surgical system (Medicaroid Corporation, Kobe, Japan), during its early clinical adoption, could achieve perioperative outcomes in robot-assisted sacrocolpopexy (RASC) for pelvic organ prolapse (POP) comparable to those of the established da Vinci® system (Intuitive Surgical, Sunnyvale, CA, USA).

Materials and methods

This single-center retrospective study included 23 patients who underwent RASC between September 2023 and December 2024. Eleven patients underwent surgery with the hinotori™ system (h-RASC), and twelve with the da Vinci® system (d-RASC). Operative time, console time, and stepwise procedural durations were evaluated.

Results

Patient characteristics were comparable between groups. Although console time showed no significant difference, operative time tended to be longer in the h-RASC group (275 vs. 242 minutes; p = 0.056), without reaching statistical significance. The time from robot roll-in to console start (16 vs. 7 minutes; p = 0.004) and posterior mesh fixation (16 vs. 12 minutes; p = 0.001) was significantly longer with the hinotori™ system. Other perioperative outcomes were similar.

Conclusion

RASC using the hinotori™ system produced comparable perioperative outcomes to those of the da Vinci® system, although some procedural delays were observed during the initial implementation period.

## Introduction

Pelvic organ prolapse (POP) is a condition in which pelvic organs such as the uterus, bladder, or rectum descend into the vagina due to weakness of the supportive pelvic tissues. Patients commonly experience symptoms such as a sensation of pelvic pressure, a vaginal bulge, urinary dysfunction, and in severe cases, pain and bleeding. These symptoms can significantly impair quality of life.

The surgical management of POP aims to restore anatomical structure and improve function. Of the various techniques, abdominal sacral colpopexy is considered the gold standard, offering superior anatomical and functional outcomes compared to transvaginal approaches [1-3]. Laparoscopic sacrocolpopexy (LSC) is a less invasive alternative associated with reduced blood loss and shorter hospitalization. However, it remains technically demanding with a steep learning curve [4,5].

Robotic-assisted sacrocolpopexy (RASC), particularly when utilizing the da Vinci® surgical system (Intuitive Surgical, Inc., Sunnyvale, California, US), was introduced to address these challenges by enhancing precise surgical procedures and reducing surgeon fatigue. Meta-analyses have shown that RASC results in lower intraoperative complication rates and comparable anatomical success to LSC, albeit with increased operative time [6,7].

The hinotori™ surgical system (Medicaroid Corporation, Kobe, Hyogo, Japan), developed and approved in 2020, introduces novel features such as eight-axis robotic arms and software-based trocar calibration [8]. These features aim to improve surgical ergonomics and efficiency. While its adoption is increasing in urological and general surgeries, data on its application in RASC remains scarce.

At our institution, RASC using the da Vinci® surgical system was introduced in October 2020, followed by the introduction of RASC using the hinotori™ surgical system in September 2023. This study aimed to evaluate the performance of the hinotori™ surgical system during its early phase of clinical adoption, specifically assessing whether it could achieve perioperative outcomes in RASC comparable to those obtained with the established da Vinci® system. Particular emphasis was placed on the stepwise analysis of procedural durations.

## Materials and methods

Study design and setting

This single-center, retrospective study was conducted at the Shiga University of Medical Science. We evaluated all consecutive patients who underwent RASC for POP at our institution between September 2023 and December 2024, without exclusions based on clinical outcome or surgeon. All procedures performed using the da Vinci® surgical system in this study utilized the da Vinci Xi® platform. Ethical approval was obtained from the Shiga University of Medical Science Review Board (Approval No. R2021-124). Informed consent was obtained in the form of an opt-out on the website of Shiga University of Medical Science Hospital. All procedures were conducted in accordance with the principles outlined in the Declaration of Helsinki.

Data collection

Patient data were collected from electronic medical records and surgical videos. This included demographic characteristics (age and body mass index), comorbidities, POP-Q stage, previous POP surgery or hysterectomy, surgical details (operation time, cockpit/console time, procedure time for each part, estimated blood loss), postoperative length of hospital stay, intra- and perioperative complications, and recurrence rates. The time of operation start was defined as the initial skin incision. The duration of each surgical step was calculated based on intraoperative recordings of the start and end timepoints, which were documented in real time according to the verbal declarations of the operating surgeon. These timepoints were subsequently verified for consistency by cross-referencing them with the corresponding surgical video recordings.

Patients

This study included a total of 23 consecutive patients who underwent RASC for POP. Of these, 11 patients underwent RASC with the hinotori™ surgical system (h-RASC group), and 12 patients had undergone RASC with the da Vinci® surgical system (d-RASC group).

Patient’s position and port placement

**Figure 1:**
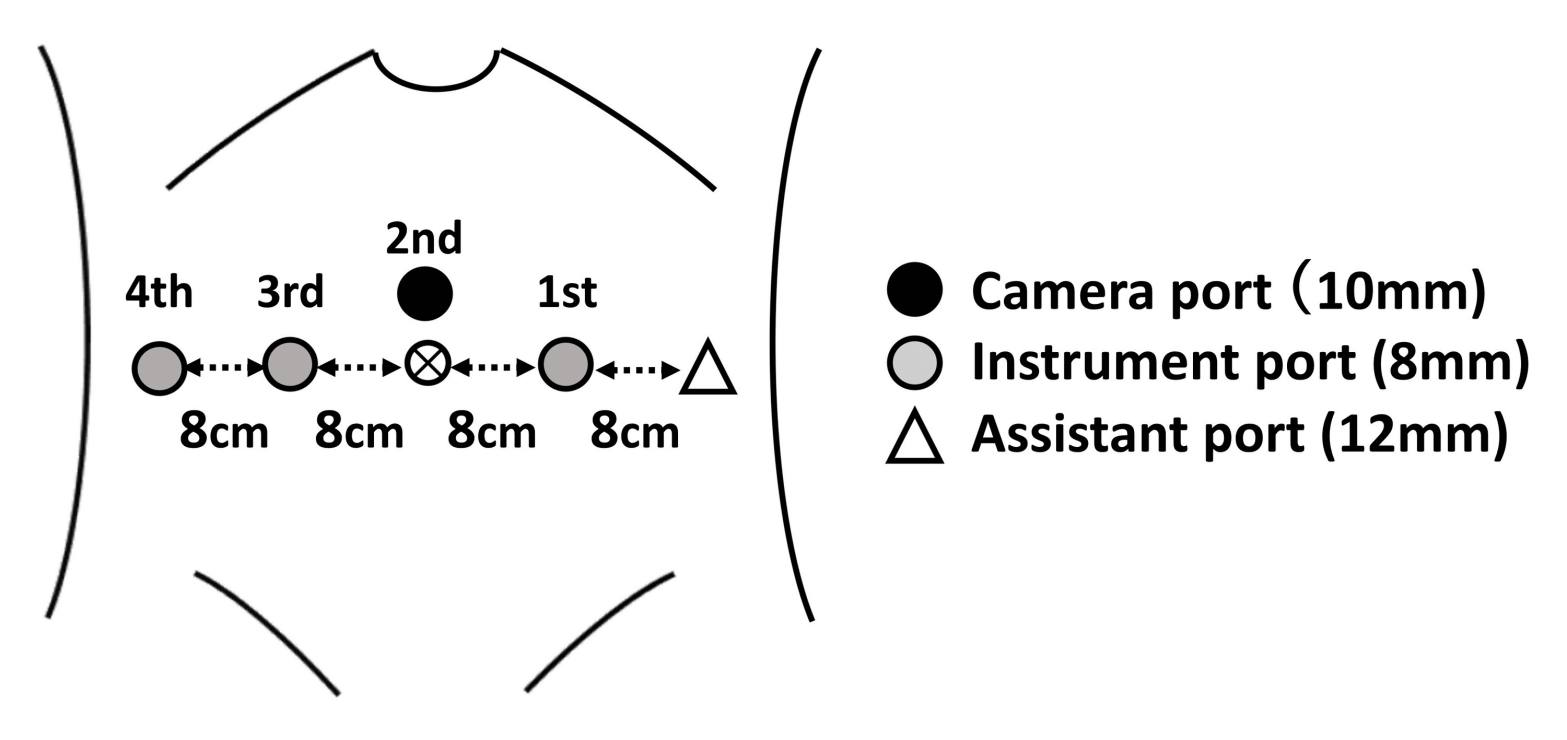
Port placement Port placement: a 10-mm camera port was placed 3 cm above the umbilicus; three 8-mm instrumentation ports (1st, 2nd, and 3rd) and one 12-mm assistant port were placed in a horizontal line around the umbilicus. The image is created by the author.

The patients were placed in the lithotomy position with a 20-25° Trendelenburg tilt. The robotic unit was positioned on the right side of the patient. Five surgical ports were placed: A 10 mm camera port was placed 3 cm above the umbilicus; three 8 mm instrumentation ports and one 12 mm assistant port were placed in a horizontal line around the umbilicus. All ports were spaced 8 cm apart (Figure 1). The instruments used in both systems are illustrated in Table 1. The hinotori™ surgical system uses the following instruments: the first port (left hand) is equipped with a bipolar fenestrated forceps, the third port (right hand) is equipped with a monopolar curved scissors and a needle holder, and the fourth port is equipped with a versatile grasping forceps. A key distinction is that the hinotori™ system lacks a needle holder with a built-in suture-cutting function, unlike the da Vinci® system.

**Table 1:** Instruments used in both surgical systems The hinotori™ surgical system uses the following instruments: the 1st port is equipped with a bipolar fenestrated forceps, the 3rd port with a monopolar curved scissors and a needle holder, and the 4th port with a versatile grasping forceps. A notable difference from the da Vinci® surgical system is that the hinotori™ system does not have a needle holder with a suture-cutting function. h-RASC: robot-assisted sacrocolpopexy with hinotori™ surgical system, d-RASC: robot-assisted sacrocolpopexy with da Vinci® surgical system

Instrument	h-RASC	d-RASC
1st	Bipolar Fenestrate	Bipolar Fenestrated
2nd	3D videoscope 0° and 30°	Stereo Endoscope 0°
3rd	Monopolar Curved scissors	Monopolar Curved scissors
	Needle Holder	Large Needle Driver Suture Cut
4th	Versatile Grasping Forceps	ProGrasp Forceps

Surgeons and surgical procedures

All robot-assisted sacrocolpopexy (RASC) procedures in this study were performed by three surgeons (designated A [S.Ku], B [T.T.], and C [R.O.]). Among the 23 RASC cases included in the study, the distribution of surgical procedures was as follows: surgeon A performed seven cases and surgeon C 4 cases using the hinotori™ surgical system, while surgeon A performed six cases, surgeon B 4 cases, and surgeon C 2 cases using the da Vinci® surgical system. Each surgical procedure was carried out by a single primary surgeon without the occurrence of intraoperative substitution.

At the commencement of the study, surgeon A had experience as a primary surgeon in ≥20 RASC procedures using the da Vinci® system, surgeon B in ≥10 procedures, and surgeon C in <5 procedures. Conversely, neither surgeon A nor surgeon C had prior clinical experience with RASC using the hinotori™ system. In order to attain proficiency with the hinotori™ platform, both surgeons underwent extensive training, including dry lab exercises, prior to performing the procedures.

**Figure 2:**
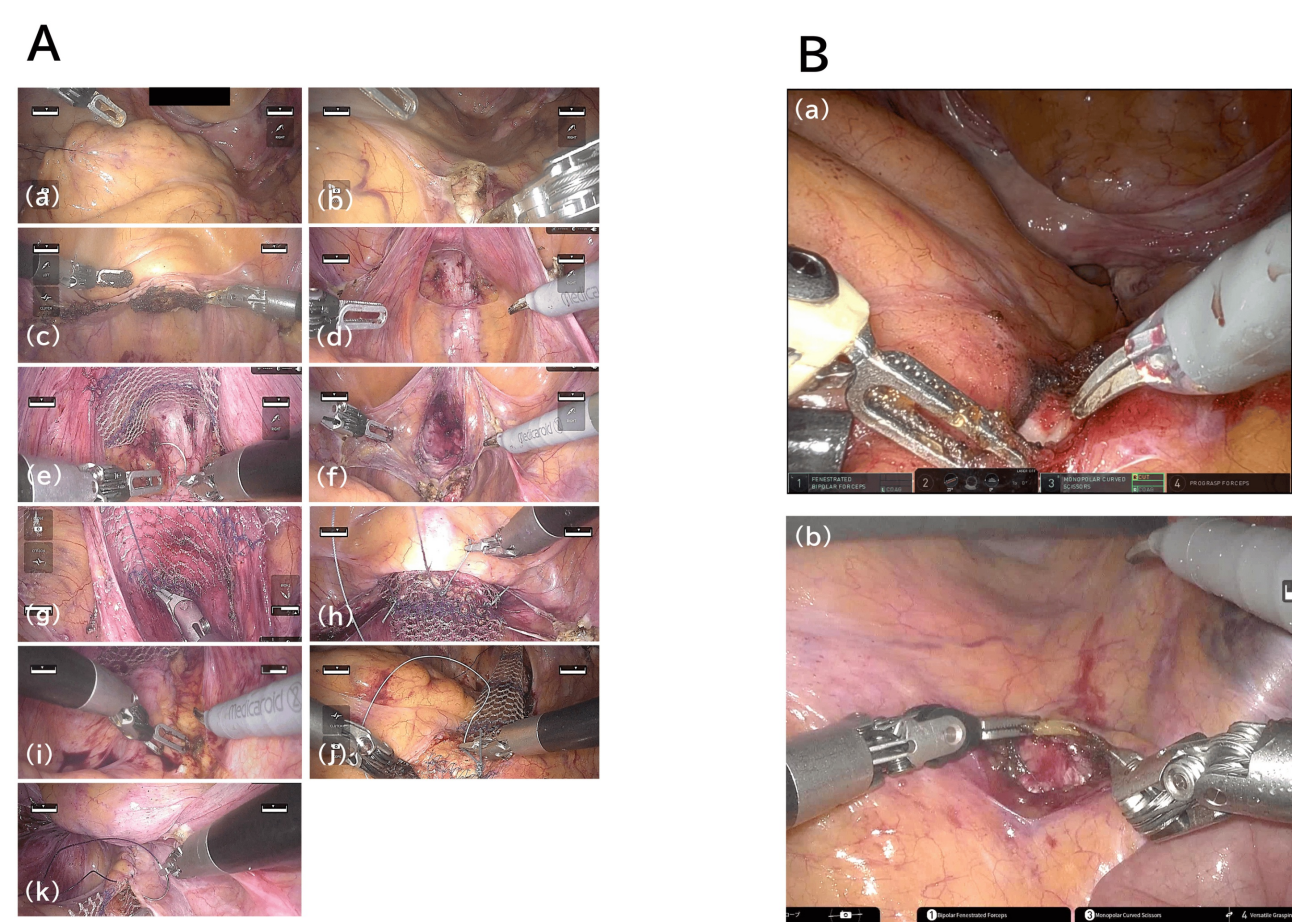
Intraoperative view of each surgical step in robot-assisted sacrocolpopexy A. Intraoperative view of each surgical step in the hinotori™ surgical system: (a) retract the sigmoid colon, (b) expose the sacral promontory, (c) subtotal hysterectomy, (d) expose posterior vaginal wall, (e) posterior mesh attachment, (f) expose the anterior vaginal wall, (g) anterior mesh attachment, (h) vaginal vault mesh attachment, (i) peritoneal incision, (j) sacral mesh attachment, and (k) peritoneal closure. B. Surgical views of the sacral promontory using (a) a 0° endoscope with the da Vinci® system and (b) a 30° videoscope with the hinotori™ system.

The surgical protocol included the following steps: lateral retraction of the sigmoid colon, exposure of the sacral promontory, subtotal hysterectomy, exposure of the posterior vaginal wall, posterior mesh attachment, exposure of the anterior vaginal wall, anterior mesh attachment, vaginal vault mesh attachment, peritoneal incision, sacral mesh attachment, and peritoneal closure (Figure 2). The same surgical protocol was followed for both robotic systems. To ensure procedural consistency, the number of stitches used for mesh fixation was standardized across both robotic systems: six stitches for anterior mesh fixation, three for posterior mesh, three for vaginal vault mesh, and two for sacral promontory fixation. For procedures involving the sacral promontory, the camera needs to be switched from a 0° scope to a 30° scope in the hinotori™ surgical system in order to adequately visualize the operative field. As shown in Figure 2B, this adjustment is necessary to obtain a surgical view comparable to that achieved with the da Vinci® system, which allows the procedure to be performed with a 0° endoscope.

Statistical analysis

Categorical variables were analyzed using the chi-square test. Continuous variables were compared using the Mann-Whitney U test. A p-value of < 0.05 was considered statistically significant. All analyses were performed using EZR (Easy R) statistical software.

## Results

The baseline characteristics of the h-RASC and d-RASC groups are presented in Table 2. There were no statistically significant differences between the two groups with respect to age, body mass index (BMI), comorbidities, type of POP, POP-Q stage, history of previous POP surgery, or hysterectomy.

**Table 2:** Patient characteristics BMI: body mass index; POP: pelvic organ prolapse; POP-Q: pelvic organ prolapse quantification; h-RASC: robot-assisted sacrocolpopexy with hinotori™ surgical system; d-RASC: robot-assisted sacrocolpopexy with da Vinci® surgical system

Variables	h-RASC group (n=11)	d-RASC group (n=12)	p-value
Median age, years (range)	76 (59-86)	74 (63-83)	0.951
Median BMI, kg/m^2^ (range)	24.2 (17.6-32.3)	22.6 (19.7-28.4)	0.356
Comorbidity, n (%)			
Hypertension	6 (54.5)	8 (66.7)	0.68
Hyperlipidemia	3 (27.2)	4 (33.3)	≥ 0.99
Diabetes mellitus	1 (9.1)	2 (16.7)	≥ 0.99
POP-Q stage, n, (%)			
≥ Ⅲ	6 (54.5)	7 (58.3)	≥ 0.99
Previous hysterectomy, n (%)	2 (18.2)	3 (25.0)	≥ 0.99
Previous POP surgery, n (%)	1 (9.1)	0 (0)	≥ 0.99
Vaginal hysterectomy with colporrhaphy	1	0	-

Table 3 provides a detailed comparison of the surgical outcomes between the two groups. No statistically significant differences were found in cockpit or console time (195 vs. 169 minutes, p = 0.169); however, there was a trend towards longer operative times in the h-RASC group (275 vs. 242 minutes, p = 0.056). Estimated blood loss and the length of hospital stay after surgery were comparable between the groups. In the d-RASC group, one postoperative complication occurred, identified as a surgical site infection. Defining recurrence as POP-Q stage II or higher prolapse, one case of recurrent prolapse was identified in each group during the follow-up period. In the d-RASC group, cystocele classified as POP-Q stage 4 was noted immediately after surgery. In the h-RASC group, the recurrence of the same type was observed at the six-month postoperative follow-up.

**Table 3:** Comparison of surgical outcomes between h-RASC and d-RASC groups h-RASC: robot-assisted sacrocolpopexy with hinotori™ surgical system; d-RASC: robot-assisted sacrocolpopexy with da Vinci® surgical system

Surgical outcomes	h-RASC group (n=11)	d-RASC group (n=12)	p-value	
				Median operation times, min (range)	275 (234-399)	242 (178-373)	0.056		
Median cockpit / console times, min (range)	195 (155-254)	169 (125-292)	0.169		
Median estimated blood loss, mL (range)	0 (0-50)	0 (0-50)	0.674		
Median postoperative hospital stay, days (range)	5 (3-8)	5.5 (4-17)	0.491		
Intraoperative complications, n (%)	0 (0)	0 (0)	≥ 0.99		
Postoperative complications, n (%)	0 (0)	1 (8.3)	0.326		
Surgical site infection	0	1	-		
Median follow-up periods, months (range)	6 (3-18)	12 (1-18)	0.134		
POP recurrence, n (%)	1 (9.1)	1 (8.3)	≥ 0.99		
Type and stage of recurrence	cystocele (POP-Q stage Ⅳ)	cystocele (POP-Q stage Ⅳ)	-		
POP: pelvic organ prolapse					

Table 4 shows a detailed comparison of procedure times for each part. The time from roll-in to the start of the cockpit or console phase was significantly longer in the h-RASC group compared to the d-RASC group (16 vs. 7 minutes, p = 0.004). During the cockpit or console phase of the procedure, the time required for posterior mesh fixation was also significantly longer in the h-RASC group (16 vs. 12 minutes, p = 0.001). In contrast, no statistically significant differences were observed between the groups for the other components of the procedure.

**Table 4:** Comparison of procedure time of each part between h-RASC and d-RASC groups h-RASC: robot-assisted sacrocolpopexy with hinotori™ surgical system; d-RASC: robot-assisted sacrocolpopexy with da Vinci® surgical system

Surgical step	h-RASC group (n=11)	d-RASC group (n=12)	p value	
				Time from operation start to cockpit or console start, min, median (range)					
Operation start to roll-in	26 (15-44)	23 (15-46)	0.497		
Roll-in to cockpit or console start	16 (8-24)	7 (2-19)	0.004*		
Time for each part during cockpit or console time, min, median (range)					
Retract sigmoid colon	6 (2-13)	5 (3-14)	0.174		
Expose sacral promontory	8 (3-15)	5 (3-36)	0.064		
Subtotal hysterectomy	35 (17-46)	33 (16-46)	0.752		
Expose posterior vaginal wall	9 (6-13)	8 (4-12)	0.620		
Posterior mesh attachment	16 (11-15)	12 (8-25)	0.001*		
Expose anterior vaginal wall	24 (13-34)	16 (8-25)	0.065		
Anterior mesh attachment	16 (14-20)	13 (12-23)	0.146		
Vaginal vault mesh attachment	16 (11-35)	12 (8-25)	0.157		
Peritoneal incision	6 (2-7)	8 (3-19)	0.085		
Sacral mesh attachment	8 (4-17)	5 (3-22)	0.228		
Peritoneal closure	24 (18-32)	27 (14-33)	0.460		
* Indicates statistical significance (*p < 0.05)					

## Discussion

The hinotori™ surgical robot system, developed by Medicaroid Corporation (Kobe, Hyogo, Japan), became the first domestically produced robotic-assisted surgical platform in Japan upon its clinical introduction in 2020 [9]. Unlike the widely established da Vinci® surgical system, which has dominated the field of robotic surgery for decades, the hinotori™ system offers distinct features that differentiate its design and functionality. While both systems utilize wristed instruments and three-dimensional visualization, early reports suggest differences in ergonomic features, the absence of haptic feedback, and the range of instrument articulation that may influence surgical performance and the learning curve [10,11].

Since its introduction, the hinotori™ system has gained traction across a variety of urologic procedures. In 2020, Hinata et al. performed the first hinotori™ -assisted radical prostatectomy for localized prostate cancer, reporting favorable perioperative outcomes, including minimal blood loss and low complication rates [8]. Subsequently, in 2023, Miyake et al. published results on hinotori™-assisted partial nephrectomy, demonstrating acceptable warm ischemia times, negative surgical margins, and preservation of renal function, comparable to outcomes reported for procedures using the da Vinci® system [12]. Furthermore, a recent systematic review and meta-analysis focusing on hinotori™-assisted radical prostatectomy reported that, although operative time and console times were longer with the hinotori™ system, other perioperative and oncological outcomes, such as positive surgical margin rates, estimated blood loss, and postoperative continence, were comparable to those achieved with established robotic platforms [13].

While evidence supporting the efficacy of the hinotori™ surgical system in procedures for prostate and renal cancers continues to accumulate, reports evaluating its application and clinical outcomes in RASC remain limited. Ichino et al. conducted the first comparative study evaluating the outcomes of an initial series of 15 RASC cases performed with the hinotori™ system, compared with 15 cases performed with the da Vinci® system within the same period. They concluded that, although operative and console times were longer with the hinotori™ system, perioperative outcomes, including complication rates and the incidence of de novo stress urinary incontinence, were comparable between the two systems. Furthermore, there were no significant differences between the groups in terms of improvements in overactive bladder symptom score and changes in uroflowmetry parameters [14].

In this study, we assessed whether the perioperative outcomes of RASC performed using the hinotori™ system, despite being in its initial clinical implementation phase, were comparable to those achieved with the well-established da Vinci® system. We specifically measured the duration of each surgical step before and during console use to assess procedural efficiency. Operative time was generally longer in the hinotori™ group (h-RASC), with a statistically significant delay observed from robot roll-in to console start. This finding is consistent with a previous study by Togami et al., which reported longer median time from roll-in to cockpit/console start with the hinotori™ surgical system during surgery for gynecologic disorders, including RASC [11]. They speculated that the limited number of cases utilizing the hinotori™ surgical system, compared to those performed with the da Vinci® system, may have amplified the influence of the surgical learning curve. Additionally, we hypothesize that the hinotori™ system’s docking-free architecture, which requires manual pivot calibration, contributes to a longer setup time compared to the automated docking mechanism employed in the da Vinci® platform.

Furthermore, our study also demonstrated that the time required for the posterior mesh fixation step was significantly longer in the h-RASC group, suggesting that certain intraoperative maneuvers may be more time-consuming with the hinotori™ system. The prolonged operative time in the posterior compartment, compared to the anterior, may be attributed to anatomical and technical factors. The posterior compartment, particularly the sacral promontory and rectovaginal space, is comparatively narrower and deeper than the anterior compartment. This anatomical configuration can pose significant challenges to visualization and access during surgical procedures. In such confined spaces, the limitation of instrument articulation and camera angle may impede the ability to maintain optimal positioning and adjust needle angles, consequently leading to repeated repositioning and extended suturing times. As demonstrated in Figure 2B, sacral promontory visualization with the hinotori™ system required switching from a 0° to a 30° endoscope, a distinction from the da Vinci® system. However, operative times for sacral promontory-related steps were comparable, indicating that equivalent procedural performance can be achieved with appropriate system-specific adjustments. Conversely, the anterior compartment offers a relatively wider and more accessible field, which may explain the absence of significant differences in operative time for that region. Fukumoto et al. reported that there was no significant difference in dissection time between the hinotori™ and da Vinci® systems. However, they noted that suturing times per stitch were longer with the hinotori™ system, likely due to ergonomic differences and early-stage familiarity with the system [15]. Specifically, they noted that the EndoWrist has a rotation range of up to 520° with the hinotori™ system, compared to 540° with the da Vinci® system. Unlike the da Vinci® system, the hinotori™ lacks a suture-cut type needle holder, meaning the assistant must cut the suture manually [15]. Differences in system performance characteristics, such as the requirement for manual pivot calibration and limited instrument articulation, may result in a longer operative time. However, it is believed that these issues can be improved with increased experience and familiarity among surgeons and assistants [16]. Our findings demonstrate that even during the early phase of implementation, the hinotori™ system achieved perioperative outcomes comparable to those of the da Vinci® system. These results are consistent with previously reported data on prostate and renal cancer surgeries using the hinotori™ system, which supports its perioperative safety profile [8,12,13,16,17]. Furthermore, a recent study suggested that the docking-free design of the hinotori™ system may reduce postoperative port-site pain compared to the da Vinci® system [18].

Nonetheless, this study has several limitations. Its retrospective design and small sample size may limit the generalizability and statistical power of the findings. Differences in surgeon experience and familiarity with the hinotori™ system, particularly during its early implementation, may have influenced operative efficiency. These operator-related factors, which were not statistically adjusted for, could explain the prolonged time required for specific steps such as posterior mesh fixation. Additionally, our statistical methods did not allow for the calculation of confidence intervals, further limiting the precision of the estimated differences.

## Conclusions

Despite being in the early phase of clinical adoption, the hinotori™ surgical system demonstrated perioperative outcomes comparable to those of the da Vinci® system in RASC, with a favorable safety profile and acceptable operative efficiency. These findings suggest that the hinotori™ system can yield surgical outcomes equivalent to the da Vinci® system in appropriately selected cases. To confirm these results and further evaluate the comparative performance of the two robotic platforms in RASC, large-scale prospective studies with balanced surgeon experience are warranted.
